# Interaction of Hydration Status and Physical Activity Level on Early Renal Damage in Children: A Longitudinal Study

**DOI:** 10.3389/fnut.2022.910291

**Published:** 2022-06-23

**Authors:** Menglong Li, Wen Shu, Nubiya Amaerjiang, Huidi Xiao, Jiawulan Zunong, Sten H. Vermund, Dayong Huang, Yifei Hu

**Affiliations:** ^1^Department of Child, Adolescent Health and Maternal Care, School of Public Health, Capital Medical University, Beijing, China; ^2^Office of the Dean, Yale School of Public Health, Yale University, New Haven, CT, United States; ^3^Department of Hematology, Beijing Friendship Hospital, Capital Medical University, Beijing, China

**Keywords:** water intake, hydration status, dehydration, physical activity, renal damage, children, longitudinal study, China

## Abstract

**Background:**

Optimal water intake positively affects various aspects of human physiology, especially renal function. Physical activity (PA) may have an impact on hydration status and renal health, but the interaction of hydration status and PA level on renal function is not well-studied in children.

**Methods:**

We conducted four waves of urine assays in our child cohort (*PROC*) study from October 2018 to November 2019 in Beijing, China. We measured urinary specific gravity, β_2_-microglobulin (β_2_-MG), and microalbumin (MA) excretion to assess hydration status and renal damage in the context of PA level and other covariates among 1,914 primary school children. We determined the associations of renal damage with the interaction of hydration status and PA level using generalized linear mixed-effects models.

**Results:**

The prevalence of dehydration was 35.0%, 62.1%, 63.9%, and 63.3%, and the prevalence of insufficient PA was 86.2%, 44.9%, 90.4%, and 90.2% from wave 1 to wave 4 among 1,914 primary school children. From wave 1 to wave 4, the prevalence of renal tubular damage had a significant increasing trend of 8.8%, 15.9%, 25.7%, and 29.0% (*Z* = 16.9, *P* < 0.001), while the prevalence of glomerular damage revealed a declining trend of 5.6%, 5.5%, 4.4%, and 4.1% (*Z* = −2.4, *P* = 0.016). There were stable longitudinal associations of renal tubular and glomerular damage with hydration status (euhydration: OR = 0.50 and 0.33, respectively) but not with PA level. In multivariate analysis, significant interactions of hydration status and PA level were noted with renal tubular damage (β = 0.43, *P* = 0.014) and glomerular damage (β = 0.60, *P* = 0.047). Children with euhydration and insufficient PA were less likely to have renal tubular damage (OR = 0.46, 95% CI: 0.39, 0.53) or glomerular damage (OR = 0.28, 95% CI: 0.20, 0.39); children with euhydration and sufficient PA were also less likely to have renal tubular damage (OR = 0.57, 95% CI: 0.44, 0.75) or glomerular damage (OR = 0.47, 95% CI: 0.30, 0.74), adjusting for age, sex, BMI z-score, standardized SBP, sleep duration, computer/cell phone screen time, and fruit and vegetable intake.

**Conclusion:**

Children with euhydration and either sufficient or insufficient PA were less likely to have early renal damage. Adequate daily water intake for children is important, especially after PA.

## Introduction

Water has been described as the “most essential” nutrient, the major constituent of the human body (1). Water intake directly affects health, and optimal water intake plays a vital role in various aspects of human physiology, especially in renal function (2–4). Global data suggest that children's water intake fails to meet recommended guidelines with the high prevalence of dehydration as a frequent consequence (5, 6). In China, high academic pressures often make students have a short inter-curriculum break; children may not drink enough fluids to reduce the micturition frequency. Due to the above behavioral habits and physiological reasons, dehydration has an adverse impact on the growth and development of children and can lead to target organ damage including cardiovascular (7) and renal (2, 8) disorders.

Healthy lifestyles promote the renal health of children, reducing long-term renal damage in adulthood (9, 10). Physical activity (PA) is a well-recognized feature of health in childhood (11, 12). However, the lack of PA among children is increasingly prevalent globally (11, 13) due to academic pressures and modern lifestyles of video games, computer and cell phone access, and urban living. PA patterns are established and modifiable early in childhood and can impact the eventual development of hypertension (14) and renal disease (15, 16).

Healthy children may have higher risk of dehydration due to higher levels of PA. Without adequate hydration, childhood renal function can be compromised, given their dynamic metabolic status (17, 18). Associations between PA and hydration status and their interactions with renal disease have been inconsistent across studies and populations (17–20). We have reported previously that dehydration status aggravated renal impairment over the school week days, notably tubular abnormalities (21). To our knowledge, there are no studies investigating the interaction of hydration status and PA level on renal damage in schoolchildren. Hence, we examined longitudinal associations of renal damage with euhydration and sufficient PA to investigate the potential interaction between hydration status and PA level on renal damage in children.

## Methods

### Study Design and Participants

**Figure 1:**
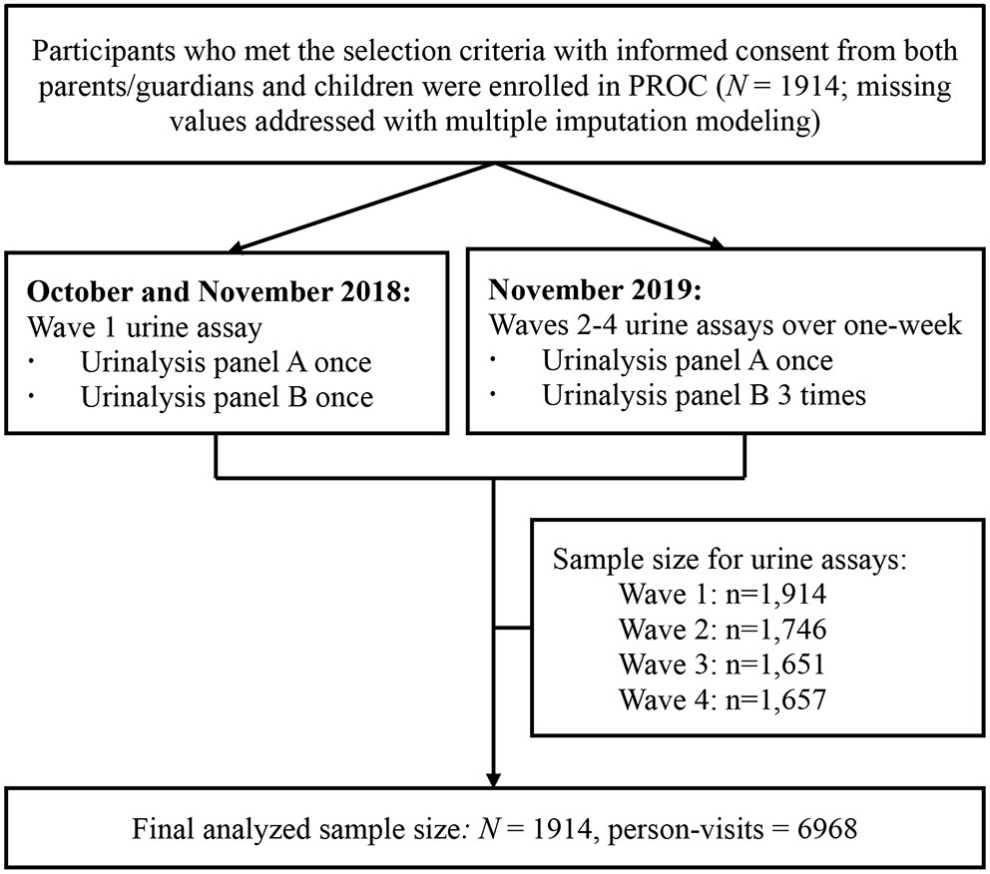
Flowchart of the procedure and wave of urine collection for the study.

The PROC study (www.chictr.org.cn/enIndex.aspx, No. ChiCTR2100044027, official website as https://www.procstudy.com) enrolled 1,914 children aged 6–8 years newly in six non-boarding primary schools in Beijing in 2018 [detailed elsewhere (22)]. All participants were recruited from the PROC cohort and were followed for four waves of repeated urine assays from October 2018 to November 2019 [detailed elsewhere (23)]. In brief, the wave 1 of urine assay was conducted at baseline and waves 2–4 of urine assays were conducted within a 1-week span periodically during the 1-year follow-up visit (Figure 1).

### Urine Measurements and Outcome Indicators

Urine collection and test procedures were detailed elsewhere (23). In brief, fasting urine assays were conducted at baseline in wave 1, 24-h urine assays were conducted from Sunday to Monday in wave 2, and fasting urine assays were conducted on Wednesday in wave 3 and Friday in wave 4. Specific gravity (SG), β_2_-microglobulin (β_2_-MG), and microalbumin (MA) were measured *via* urinalysis panels A and B. Urinalysis panel A includes urobilinogen, bilirubin, ketone, occult blood, protein, nitrite, white blood cells, sugar, SG, pH, color, and turbidity; urinalysis panel B includes β_2_-MG, microalbumin, potassium, sodium, uric acid, and creatinine. Dehydration status of participants was defined as SG ≥ 1.020, with euhydration status defined as SG < 1.020 (4, 21). Renal tubular damage was defined as elevated β_2_-MG > 0.2 mg/L (24), and glomerular damage was defined as elevated MA ≥20 mg/L (25).

### Data Collection of Covariates

Anthropometric measurements were conducted by trained staff and included height, weight, BMI, and blood pressure [detailed elsewhere (23)]. Z-scores of height, weight, and body mass index (BMI) were calculated using 2007 WHO standards and standardized SBP was calculated by age and sex group. Lifestyle information were reported by parents using self-administrated questionnaires, including sleep duration (Children's Sleep Habits Questionnaire [CSHQ]) (26, 27), fruit and vegetable intake (FVI; 16-item Mediterranean Diet Quality Index in children and adolescents [KIDMED]) (23, 28), and computer/cell phone screen time and PA time (self-administrated questionnaire, including 17 activities indoor and outdoor lasting at least 15 min, based on Children's Leisure Activities Study Survey Chinese version [CLASS-C]) (29). Short sleep for children was defined as sleep duration <10 h/day. Insufficient FVI was defined as <4/day. Long screen time was defined as computer/cell phone screen time ≥2 h/day. PA level as the main lifestyle variate was reported daily by parents for 1 week and was calculated both as a weekly average (total PA time/7 days) and as weekly patterns (week-day PA time/5 days and weekend PA time/2 days). PA levels were linked to the date of the urinalysis. Insufficient PA was defined as <1 h/day with weekend and weekday urinalysis: Monday tests linked to the PA level over the weekend, and Tuesday–Friday weekday tests linked to the PA level during that weekday school period.

### Statistical Analysis

Descriptive statistics are presented by study wave. Categorical variables such as sex or lifestyle covariates are presented as counts and percentages. Continuous variables such as height z-score, weight z-score, or BMI z-score are described as the mean ± standard deviation (SD). Multiple imputations were performed for variables with missing values and 50 complete datasets were obtained for analysis. Trend χ^2^ tests were performed to determine the prevalence trend by the study waves. Generalized linear mixed-effects models were generated to determine the associations and odds ratio (OR) with 95% confidence interval (95% CI) of renal damage with the direct association and interaction of hydration status and PA level, while the week-day and intra-wave of the urinalysis were included as random effects. The values in Table 1 were calculated based on the first imputed dataset, while statistical inferences of the parameters in Tables 2–**4** were performed on 50 datasets using PROC MIANALYZE. A two-tailed *P*-value of 0.05 was used to define statistical significance. All data were analyzed using Statistical Analysis System V.9.4 (SAS Institute Inc., Cary, North Carolina, USA).

**Table 1:** Demographic characteristics among 6–9-year-old children, Beijing, China (*N* = 1914).

**Characteristics**	**Wave 1 (*n* = 1,914)**	**Wave 2 (*n* = 1,746)**	**Wave 3 (*n* = 1,651)**	**Wave 4 (*n* = 1,657)**
Age (year)^a^	6.6 ± 0.3	7.6 ± 0.3	7.6 ± 0.3	7.6 ± 0.3
Sex, boys^b^	956 (50.0)	875 (50.1)	820 (49.7)	826 (49.8)
Height z-score^a^	0.67 ± 0.96	0.79 ± 0.97	0.77 ± 0.97	0.77 ± 0.97
Weight z-score^a^	0.70 ± 1.41	0.99 ± 1.48	0.98 ± 1.48	0.98 ± 1.46
Body mass index (BMI) z-score^a^	0.40 ± 1.54	0.70 ± 1.59	0.69 ± 1.59	0.70 ± 1.57
Systolic blood pressure (SBP in mmHg)^a^	101 ± 8	101 ± 8	101 ± 9	101 ± 8
Diastolic blood pressure (DBP in mmHg)^a^	56 ± 6	56 ± 6	56 ± 6	56 ± 6
Short sleep (<10 h/d)^b^	1,441 (75.3)	1,327 (76.0)	1,246 (75.5)	1,249 (75.4)
Long screen time (≥2 h/d)^b^	95 (5.0)	86 (4.9)	79 (4.8)	80 (4.8)
Insufficient fruit/vegetable intake (FVI <4 /d)^b^	931 (48.6)	864 (49.5)	813 (49.2)	818 (49.4)
Insufficient physical activity (PA weekly average <1 h/d)^b^	1,451 (75.8)	1,323 (75.8)	1,259 (76.3)	1,267 (76.5)
Insufficient PA (weekly patterns <1 h/d)^b^	1,649 (86.2)	784 (44.9)	1,493 (90.4)	1,495 (90.2)
Dehydration status (specific gravity ≥1.02)^b^	670 (35.0)	1,085 (62.1)	1,055 (63.9)	1,049 (63.3)
Renal tubular damage (β_2_-MG >0.2 mg/L)^b^	168 (8.8)	277 (15.9)	425 (25.7)	481 (29.0)
Renal glomerular damage (MA ≥20 mg/L)^b^	107 (5.6)	96 (5.5)	72 (4.4)	68 (4.1)

*β_2_-MG, β_2_-microglobulin; MA, microalbumin*;

a *Mean and standard deviation*;

b *n (%)*.

**Table 2:** Bivariate associations of renal damage with hydration status and physical activity level using generalized linear mixed-effects models among 6–9-year-old children in Beijing (*N* = 1914).

**Dependent variables**	**Independent variables**	**Model 1**		**Model 2**		**Model 3**	
		**cOR (95%CI)**	***P***	**aOR (95%CI)**	***P***	**aOR (95%CI)**	***P***
Tubular damage	Dehydration	ref.		ref.		ref.	
	Euhydration	0.52 (0.46, 0.59)	<0.001	0.50 (0.44, 0.57)	<0.001	0.50 (0.44, 0.57)	<0.001
Tubular damage	Insufficient PA	ref.		ref.		ref.	
	Sufficient PA	0.94 (0.78, 1.13)	0.51	0.94 (0.78, 1.13)	0.50	0.93 (0.77, 1.12)	0.47
Glomerular damage	Dehydration	ref.		ref.		ref.	
	Euhydration	0.36 (0.28, 0.47)	<0.001	0.32 (0.25, 0.42)	<0.001	0.33 (0.25, 0.43)	<0.001
Glomerular damage	Insufficient PA	ref.		ref.		ref.	
	Sufficient PA	1.12 (0.84, 1.50)	0.44	1.14 (0.84, 1.53)	0.40	1.15 (0.85, 1.55)	0.35

*PA, physical activity; cOR, crude odds ratio; aOR, adjusted odds ratio; CI, confidence interval; ref., reference group; Model 1, unadjusted; model 2, adjusting for age, sex, and BMI z-score; model 3, adjusting for age, sex, BMI z-score, standardized SBP, sleep duration, screen time, and fruit and vegetable intake. All models included two random effects, namely, the week-day and intra-wave of the urinalysis*.

## Results

### Demographic Characteristics

From the PROC cohort, 1,914 participants aged 6.6 ± 0.3 years were enrolled, half boys and half girls, with 87% retention through wave 4 (Table 1). The height *z*-score, weight *z*-score, and BMI *z*-score suggested appropriate nutritional status as would be expected in a general pediatric population. The SBP and DBP was 101 ± 8 and 56 ± 6 mmHg, respectively. The prevalence of short sleep, long screen time, and insufficient FVI was 75.3%, 5.0%, and 48.6%, respectively. The prevalence of dehydration was 35.0%, 62.1%, 63.9%, and 63.3%, and the prevalence of insufficient PA for weekly patterns was 86.2%, 44.9%, 90.4% and 90.2% from waves 1–4. An increased trend for tubular damage was noted for waves 1–4 (8.8%, 15.9%, 25.7%, and 29.0%; *Z* = 16.9, *P* < 0.001), while glomerular damage showed a decreasing trend (5.6%, 5.5%, 4.4%, and 4.1%; *Z* = −2.4, *P* = 0.016; Table 1).

### Binary Associations of Renal Damage With Hydration Status and Physical Activity

Stable longitudinal associations of renal tubular damage with hydration status were observed, but no association was seen with PA level (weekly patterns) in unadjusted model 1 and model 2 adjusting for age, sex, and BMI. Children with euhydration were less likely to have renal tubular damage (OR = 0.50, 95% CI: 0.44, 0.57) adjusting for age, sex, BMI *z*-score, standardized SBP, sleep duration, screen time, and FVI (model 3; Table 2). Stable longitudinal associations of renal glomerular damage with hydration status were observed in unadjusted model 1 and adjusted model 2. Children with euhydration were less likely to have renal glomerular damage (OR = 0.33, 95% CI: 0.25, 0.43) adjusting for age, sex, BMI z-score, standardized SBP, sleep duration, screen time, and FVI (model 3; Table 2).

### Multivariable Associations of Renal Damage With Hydration Status and Physical Activity

More extensive multivariable analyses showed consistent results with binary analysis that children with euhydration were less likely to have renal tubular damage (β = −0.78, 95% CI: −0.93, −0.63; *P* < 0.001) and renal glomerular damage (β = −1.27, 95% CI: −1.59, −0.95; *P* < 0.001) adjusting for age, sex, BMI z-score, standardized SBP, sleep duration, screen time, and FVI (model 3; Table 3). Moreover, we observed significant interaction of hydration status and PA level (weekly patterns) on renal tubular damage in adjusted model 3 (β = 0.43, *P* = 0.014) and on renal glomerular damage in unadjusted model 1 (β = 0.60, *P* = 0.047; Table 3).

**Table 3:** Multivariable associations of renal damage with hydration status and physical activity level using generalized linear mixed-effects models among 6–9-year-old children in Beijing (*N* = 1914).

**Dependent variables**	**Independent variables**	**Model 1**		**Model 2**		**Model 3**	
		**Estimate (95%CI)**	***P***	**Estimate (95%CI)**	***P***	**Estimate (95%CI)**	***P***
Tubular	Intercept	−1.16 (−1.72, −0.61)	<0.001	−2.31(−3.89, −0.73)	0.004	−2.15 (−3.74, −0.56)	0.008
damage	Euhydration	−0.75 (−0.90, −0.59)	<0.001	−0.78 (−0.93, −0.63)	<0.001	−0.78 (−0.93, −0.63)	<0.001
	Sufficient PA	−0.21 (−0.43, 0.01)	0.066	−0.20 (−0.42, 0.02)	0.074	−0.21 (−0.43, 0.02)	0.069
	Interaction	0.45 (0.11, 0.79)	0.010	0.44 (0.09, 0.78)	0.012	0.43 (0.09, 0.77)	0.014
Glomerular	Intercept	−2.61 (−2.91, −2.30)	<0.001	−3.21 (−5.80, −0.63)	0.015	−2.98 (−5.56, −0.40)	0.024
damage	Euhydration	−1.18 (−1.49, −0.87)	<0.001	−1.28 (−1.60, −0.96)	<0.001	−1.27 (−1.59, −0.95)	<0.001
	Sufficient PA	−0.08 (−0.44, 0.28)	0.67	−0.06 (−0.43, 0.31)	0.76	−0.05 (−0.42, 0.32)	0.80
	Interaction	0.60 (0.01, 1.19)	0.047	0.57 (−0.03, 1.17)	0.060	0.57 (−0.04, 1.17)	0.065

*PA, physical activity; CI, confidence interval; Model 1, unadjusted; model 2, adjusting for age, sex, and BMI z–score; model 3, adjusting for age, sex, BMI z–score, standardized SBP, sleep duration, screen time, and fruit and vegetable intake. All models included two random effects, namely, the week–day and intra–wave of the urinalysis*.

### Interaction of Hydration Status and Physical Activity Level on Renal Damage

Taking children with dehydration and insufficient PA (weekly patterns) as reference, renal tubular damage was less likely to happen among those with euhydration and insufficient PA (OR = 0.46, 95% CI: 0.39, 0.53) or with euhydration and sufficient PA (OR = 0.57, 95% CI: 0.44, 0.75), adjusting for age, sex, BMI z-score, standardized SBP, sleep duration, screen time, and FVI (model 3; Table 4). Renal glomerular damage was less likely to happen among those with euhydration and insufficient PA (OR = 0.28, 95% CI: 0.20, 0.39) or with euhydration and sufficient PA (OR = 0.47, 95% CI: 0.30, 0.74), adjusting for age, sex, BMI z-score, standardized SBP, sleep duration, screen time, and FVI (model 3; Table 4).

**Table 4:** Interaction of hydration status and physical activity level on renal damage using generalized linear mixed–effects models among 6–9–year–old children in Beijing (*N* = 1914).

**Dependent variables**	**Independent variables**	**Model 1**		**Model 2**		**Model 3**	
		**cOR (95%CI)**	***P***	**aOR (95%CI)**	***P***	**aOR (95%CI)**	***P***
Tubular	Dehydration + Insufficient PA	ref.					
damage	Dehydration + Sufficient PA	0.81 (0.65, 1.01)	0.066	0.82 (0.66, 1.02)	0.074	0.81 (0.65, 1.02)	0.069
	Euhydration + Insufficient PA	0.47 (0.41, 0.55)	<0.001	0.46 (0.39, 0.53)	<0.001	0.46 (0.39, 0.53)	<0.001
	Euhydration + Sufficient PA	0.60 (0.46, 0.79)	<0.001	0.58 (0.44, 0.76)	<0.001	0.57 (0.44, 0.75)	<0.001
Glomerular	Dehydration + Insufficient PA	ref.					
damage	Dehydration + Sufficient PA	0.93 (0.65, 1.33)	0.67	0.94 (0.65, 1.37)	0.76	0.95 (0.66, 1.38)	0.80
	Euhydration + Insufficient PA	0.31 (0.22, 0.42)	<0.001	0.28 (0.20, 0.38)	<0.001	0.28 (0.20, 0.39)	<0.001
	Euhydration + Sufficient PA	0.52 (0.33, 0.80)	0.004	0.47 (0.30, 0.73)	0.001	0.47 (0.30, 0.74)	0.001

*PA, physical activity; cOR, crude odds ratio; aOR, adjusted odds ratio; CI, confidence interval; ref., reference group; Model 1, unadjusted; model 2, adjusting for age, sex, and BMI z–score; model 3, adjusting for age, sex, BMI z–score, standardized SBP, sleep duration, screen time, and fruit and vegetable intake. All models included two random effects: the week–day and intra–wave of the urinalysis*.

## Discussion

Our study used longitudinal data from 1,914 children aged 6–9 years to assess the association between hydration status, PA level, and early renal damage in a general pediatric population in China. Overall prevalence of dehydration was 35% in children newly enrolled in elementary school and 63% when they experienced 1 year of schooling. We found that children with euhydration and sufficient PA were less likely to have early renal damage, controlling for key covariates, including age, sex, BMI, SBP, sleep duration, screen time, and FVI. A novel finding is the significant interaction of hydration status and PA level in terms of both tubular and glomerular renal damage. Children with euhydration and sufficient PA were 43% less likely to have tubular damage and 53% less likely to have glomerular damage. However, this was similar in children with euhydration and insufficient PA who were 54% less likely to have tubular damage and 72% less likely to have glomerular damage, presenting a slightly lower risk than in children with sufficient PA with the same euhydration status. These findings underscore the primary necessity of adequate water intake during PA and daily life to prevent early renal damage in schoolchildren.

The dramatic increased prevalence of dehydration (determined by urine SG) among children aged 6.6 ± 0.3 years at baseline from 35% to 63% at 1 year follow-up is notable. Similar with our follow-up prevalence, another study suggested a similar two-thirds prevalence of dehydration among Chinese children and adolescents (4). A systematic review focusing on water intake and hydration state in children reported that 60% ± 24% of children from 19 countries failed to meet the guidelines of water/ fluid intake recommended by the U.S. Institute of Medicine (IOM), European Food Safety Authority (EFSA), and Chinese and Indonesian health authorities (5). The difference in hydration between those newly enrolled and 1-year later may be due to unfavorable school environment with inadequate water access and limited time for drinking during and between classes (6).

Water intake is associated with cognition of children (30). Children's subjective feeling of thirst is not well correlated with fluid intake and this can lead to dehydration (31). A cross-sectional study of 141 adolescents aged 15–17 years reported that 90% of were dehydrated during school as determined by urine SG (32). Dehydration and inadequate water intake can affect the school performance including alertness, concentration, and fatigue (32), can impair renal function (21), and can even lead to chronic kidney disease (2). We observed stable and consistent longitudinal associations between hydration status and renal stress; children with euhydration were less likely to have tubular or glomerular damage. This finding is consistent with our previous study (21). Moreover, a decreasing temporal trend over the school week of MA only in the children with euhydration (21). Almost all available evidence support that we should promote adequate water intake, especially among students in elementary schools.

The prevalence of insufficient PA among our participants was about 76% *via* the estimate of weekly average <1 h/day, similar with the result of the National Survey of Children's Health 2017–2018 reported that about 22.6% Chinese children and adolescent had 60 min of physical activity every day during the past week (33). The prevalence of insufficient PA estimated *via* weekly patterns was about 45% at weekend and 90% at week-day, and similar trend of weekend PA level was more than week-day was reported in a cross-sectional study among 15,203 children aged 6–12 years in China (14). Different from other studies (16, 34–37), we did not observe longitudinal associations between PA level (weekly patterns) and renal damage, similar with an interventional study among obese boys in Portugal (38).

We observed a significant interaction of hydration status and PA level on both tubular damage and glomerular damage. Our generalized linear mixed-effects models including this interaction term showed that children with euhydration and sufficient PA were 43% less likely to have tubular damage and 53% less likely to have glomerular damage. Children with euhydration and insufficient PA were 54% less likely to have tubular damage and 72% less likely to have glomerular damage, presenting a higher risk than seen with sufficient PA in the same euhydration status. Few studies focus on the interaction of hydration status and PA level on renal damage in adult populations (39–41), and we have found no prior longitudinal study conducted in children. One study on healthy male adults reported that the renal function (estimated glomerular filtration rate, eGFR) did not change after acute exercise, whereas it significantly decreased after prolonged exercise, suggested that prolonged physical activity without proper hydration could be a risk factor of renal function impairment (39). We hypothesize that insufficient PA may be renal-protective compared to sufficient PA in euhydration status. A cross-sectional study among 242 Spanish school children aged 8.9 ± 1.2 years reported that PA level (practice ≥1 h/day) was associated with a higher risk of dehydration status (OR = 1.75), adjusting for sex and other lifestyle factors (17), suggest that increased PA may lead to dehydration. A study examined renal circadian rhythm in obese adolescents, after conducting dietary restriction, increased PA, and psychological support among 34 adolescents (mean age 15.7 years), the investigators observed lower diurnal free water clearance compared with nocturnal values (42), suggesting transient renal stress from diurnal PA. A trial found that the risk for acute kidney injury (AKI) is higher in participants with greater hyperthermia and dehydration during physical work; alleviating hyperthermia and/or limiting dehydration equally reduced AKI risk (40). One trial enrolled 14 men to study hypohydration caused by physical work and found that increased renal injury happened at the proximal tubules (41). This is consistent with our study using β_2_-MG excretion to estimate proximal tubular function. We further observed a combined effect of euhydration and PA level in terms of glomerular damage. We conclude that to prevent renal damage and potential functional impairment in children, optimized daily water intake, especially after PA, is needed.

The major strength of this study was the use of longitudinal urinalysis data of a general healthy pediatric population in China with a large sample size. Our use of imputation methods for missing data can reduce bias (43). Furthermore, we used linear mixed-effects models and chose key covariates to adjusted for associations of hydration status and PA level with renal damage, especially SBP and lifestyle factors such as sleep duration, screen time, and FVI. However, our study was limited by not considering other renal function indicators or biomarkers. Urine β_2_-MG, MA were tested *via* different machines due to the limited capacity of individual testing sites within allowed condition for sample processing. Hydration status was assessed using the SG of morning urine, which may overestimate the prevalence of dehydration. Moreover, hydration status and renal damage may be transient (44), and the result may only represent the situation at the time being of test and survey. We sought to minimize bias from these effects with random effects modeling with longitudinal data.

## Conclusion

We have found longitudinal interactions of hydration status and PA level on early renal damage and have found increased dehydration among the children over time in China. We found that children can be protected from early renal damage by euhydration, either with sufficient or insufficient PA. Our findings underscore the necessity of advocating adequate water intake, especially after PA, to prevent potential function impairment in healthy children and possible utilization among those with compromised renal function, especially with CKD.

## Data Availability Statement

The original contributions presented in the study are included in the article/supplementary material, further inquiries can be directed to the corresponding author.

## Ethics Statement

The studies involving human participants were reviewed and approved by Ethics Committee of Capital Medical University. Written informed consent to participate in this study was provided by the participants' legal guardian/next of kin.

## Author Contributions

YH, DH, and ML conceptualized and designed this study. ML, WS, HX, and JZ carried out the survey. DH read and reported the clinical significance of the assay. ML performed statistical analysis of the data. NA checked data analysis process. ML and WS drafted the manuscript. SV, DH, and YH edited, helped interpret, and revised the manuscript. All authors were involved in writing the study and had final approval of the submitted and published versions.

## Funding

This study was funded by the National Natural Science Foundation of China (YH, Grant No. 82073574), the Beijing Natural Science Foundation (YH, Grant No. 7202009), and the Capital's Funds for Health Improvement and Research (YH, Grant No. 2022-1G-4262).

## Conflict of Interest

The authors declare that the research was conducted in the absence of any commercial or financial relationships that could be construed as a potential conflict of interest.

## Publisher's Note

All claims expressed in this article are solely those of the authors and do not necessarily represent those of their affiliated organizations, or those of the publisher, the editors and the reviewers. Any product that may be evaluated in this article, or claim that may be made by its manufacturer, is not guaranteed or endorsed by the publisher.
